# Sustainability of hand hygiene compliance in a crowded emergency and trauma setting within a public tertiary healthcare facility

**DOI:** 10.1017/ash.2025.130

**Published:** 2025-09-03

**Authors:** Peh Yee Lee, Jiancong Wang, Siti Nur Zulydiana Binti Abang Ahmad, Alicia Ak Juan, Yew Fong Lee

**Affiliations:** 1 Infection Prevention and Control UnitSarawak General Hospital, Ministry of Health, Malaysia; 2 Institute of Biometry and Epidemiology, The German Diabetes Center, Heinrich Heine University Düsseldorf, Düsseldorf, Germany; 3 Deputy Director’s Office, Sarawak General HospitalMinistry of Health, Malaysia; 4 School of Medical and Life SciencesSunway University, Kuala Lumpur, Malaysia; 5 Emergency and Trauma DepartmentSarawak General Hospital, Ministry of Health, Malaysia

## Abstract

**Introduction:** Crowded Emergency and Trauma Department (ETD) have been associated with adverse patient outcomes and higher mortality rates. Crowding and lack of alcohol-based hand rubs (ABHRs) have been found to correlate with lower compliance to hand hygiene (HH) protocols among healthcare workers (HWs). This project aimed to improve and sustain HH compliance (HHC) among HWs in the ETD by adapting to the World Health Organization (WHO) HH Multimodal Improvement Strategy. **Methodology:** This is a cross-sectional study in ETD, Sarawak General Hospital, a university-affiliated, public tertiary-care hospital in Malaysia. It spanned 12 months, from Jan 2023 to Jan 2024. The intervention involved installing wall-mounted automated ABHR dispensers at multiple fixed locations in ETD. Pre-, during, and post-12 weeks intervention HHC audit were conducted according WHO’s gold-standard direct observation method. We conducted a sequential trend analysis and compared proportions across these periods using a linear logistic regression model to assess the improvement and sustainability of HHC. **Results & Discussion:** Mean HHC improved from 66% (383/579) (95% confidence interval [CI], 62.1%-70.0%) in the pre- intervention period to 81% (321/397) (95% CI, 76.6%-84.6%) in the intervention period, and further sustained at 85% (302/352) (95% CI, 81.7%-89.3%) in the post-intervention period (P value<0.05). The positive coefficient of 1.13 in the model, when moving from the pre- to the post-intervention period indicates a positive trend in HH compliance. The availability of adequate wall-mounted automated ABHR dispensers at multiple fixed locations at ETD created easy accessibility of ABHRs for HWs and acted as visual reminders for good HH behavior at the ETD. **Conclusions:** Having wall-mounted automated ABHR dispensers in various fixed locations proved effective in promoting good HH among HWs in emergency settings. It’s essential to have fixed ABHR dispenser placement in crowded - areas like the ETD to improve and sustain HHC among HWs.

